# Making the Bridge Between Experiment and Theory in Metal Oxides for Renewable Energy: Based on TiO_2_, ZnO, and BiVO_4_

**DOI:** 10.3390/ijms27052087

**Published:** 2026-02-24

**Authors:** Habtamu F. Etefa, Francis B. Dejene

**Affiliations:** Department of Physics, Walter Sisulu University, Private Bag X-1, Mthatha 5117, South Africa; dr.habtamu@dadu.edu.et

**Keywords:** experimental, DFT, renewable energy, metal oxide, machine learning

## Abstract

Metal oxides such as TiO_2_, ZnO, and BiVO_4_ have emerged as pivotal materials for renewable energy technologies owing to their versatile electronic, optical, and catalytic properties. This review highlights the importance of bridging experimental investigations with Density Functional Theory (DFT) to deepen the understanding of structure–property relationships in these systems. Experimental approaches provide critical insights into synthesis strategies, performance evaluation, and sustainability, whereas DFT offers predictive power at the atomic scale by elucidating electronic structures, reaction mechanisms, and defect dynamics. The synergy of these methods enables the rational design of advanced materials for photocatalysis, solar cells, and energy storage applications. Looking ahead, research opportunities lie in the development of doped and heterostructured metal oxides, the integration of machine learning for accelerated material discovery, and the implementation of in situ/operando studies that capture time-resolved phenomena. By making the bridge between experiment and theory, significant progress can be achieved toward sustainable and efficient energy solutions.

## 1. Introduction

The global energy landscape is undergoing a profound transformation driven by the escalating demand for energy, concerns over climate change, and the urgent need for sustainable development [1,2]. As the world population grows and economies continue to expand, energy consumption is projected to increase significantly by more than 25% by 2040, according to the International Energy Agency (IEA) [3,4]. Despite technological advances, the global energy system remains heavily reliant on fossil fuels, which account for over 70% of energy-related CO_2_ emissions [5,6]. This dependence poses serious environmental threats, including global warming, extreme weather events, rising sea levels, and ecosystem degradation [7]. At the same time, energy poverty persists in many regions, particularly in Sub-Saharan Africa and South Asia [8,9], where millions of people still lack access to electricity and clean cooking solutions [10]. Addressing this dual challenge reducing carbon emissions while ensuring universal access to affordable and reliable energy has become a central goal of international energy policy and is encapsulated in the United Nations Sustainable Development Goal 7 (SDG7) [11,12].

In response, there is a global push toward green and renewable energy technologies such as solar, wind, hydro, and bioenergy. Among these, solar energy stands out due to its abundance and scalability. However, the widespread deployment of green energy systems is hindered by issues such as intermittency, low efficiency, limited durability of materials, and high production costs [5]. Overcoming these limitations requires breakthroughs in materials science, particularly in the design and development of efficient, stable, and earth-abundant materials for energy conversion and storage.

Metal oxides have emerged as a critical class of materials in this context due to their favorable properties chemical stability [13,14], tunable band structures, non-toxicity, and natural abundance. They play essential roles in a wide range of energy-related applications, including photocatalysis, solar energy harvesting, batteries, supercapacitors, and hydrogen production via water splitting [6]. To optimize the performance of these materials, researchers have increasingly adopted a dual approach that integrates experimental investigations with theoretical modeling, particularly Density Functional Theory (DFT). Experimental techniques provide insight into structural, optical, and electronic properties and validate material performance under real-world conditions [15]. In contrast, DFT offers atomistic-level understanding of electronic structures, defect dynamics, adsorption energies, and reaction mechanisms, enabling the rational design and screening of new materials before synthesis [7,8]. By combining experimental data with DFT simulations, researchers can uncover the fundamental structure–property relationships that govern energy conversion processes. This synergistic strategy accelerates the development of next-generation metal oxide materials tailored for high-efficiency green energy technologies.

Green energy technologies play a critical role in mitigating climate change, enhancing energy security, and promoting sustainable development by reducing dependence on fossil fuels and lowering greenhouse gas emissions [16]. These technologies such as solar photovoltaics, wind turbines, bioenergy, and hydropower offer renewable, clean alternatives that support the transition to a low-carbon economy while stimulating job creation and technological innovation [17]. Their deployment contributes significantly to achieving global climate targets set by the Paris Agreement and supports the United Nations Sustainable Development Goals, particularly Goal 7 (Affordable and Clean Energy) and Goal 13 (Climate Action). By decentralizing energy production and enabling access in remote areas, green technologies also foster social equity and resilience in energy systems [11].

This study provides a comprehensive overview of recent advances in metal oxide materials for green energy applications, highlighting insights gained through experimental techniques and DFT simulations. The discussion focuses on how this combined approach informs the design, optimization, and mechanistic understanding of metal oxides in key applications such as photocatalysis, solar energy conversion, energy storage, and hydrogen evolution. The review aims to bridge the gap between theoretical predictions and experimental realizations, guiding the development of efficient and sustainable solutions for the global energy transition.

## 2. Metal Oxides in Renewable Energy Applications

In recent years, synergy between density functional theory (DFT) and experimental investigations has unlocked deeper mechanistic understanding and performance gains in metal-oxide systems for renewable-energy applications. For instance, a ZnO–TiO_2_ heterojunction assembled via electrostatic self-assembly was studied both experimentally and via DFT, revealing formation of a type-II interface that suppresses charge recombination and enhances photocatalytic activity resulting in 3.5-fold higher photodegradation rates and remarkable photocurrent densities in water-splitting tests [18]. In another case, DFT elucidated how substitutional N-doping at specific lattice sites in TiO_2_(B) reduces bandgap and lowers hydrogen adsorption reaction energies, thereby supporting improved electrocatalytic hydrogen evolution [19]. Likewise, in BiVO_4_ photoanodes, DFT combined with experimental defect-engineering studies demonstrated that carefully distributed (rather than clustered) oxygen vacancies mitigate charge recombination by avoiding trap-centre formation substantially enhancing photoelectrochemical water oxidation activity [20].

### 2.1. Photocatalysis (e.g., Water Splitting, Pollutant Degradation)

Metal oxides such as TiO_2_, ZnO, and BiVO_4_ are pivotal in green energy applications, notably in photocatalysis for water splitting and pollutant degradation. Their effectiveness hinges on factors like light absorption, charge transport, and surface defects. Below Table 1 is a comparative overview of these materials:

TiO_2_ has excellent stability, its wide bandgap limits visible light absorption. Techniques like doping and forming heterojunctions with materials like BiVO_4_ can enhance its photocatalytic performance. ZnO shares similarities with TiO_2_ but is more susceptible to photocorrosion. Incorporating defects and forming composites can improve its activity under visible light. BiVO_4_ naturally absorbs visible light but suffers from poor charge transport. Combining it with materials like TiO_2_ or adding co-catalysts can mitigate this issue.

**Figure 1:**
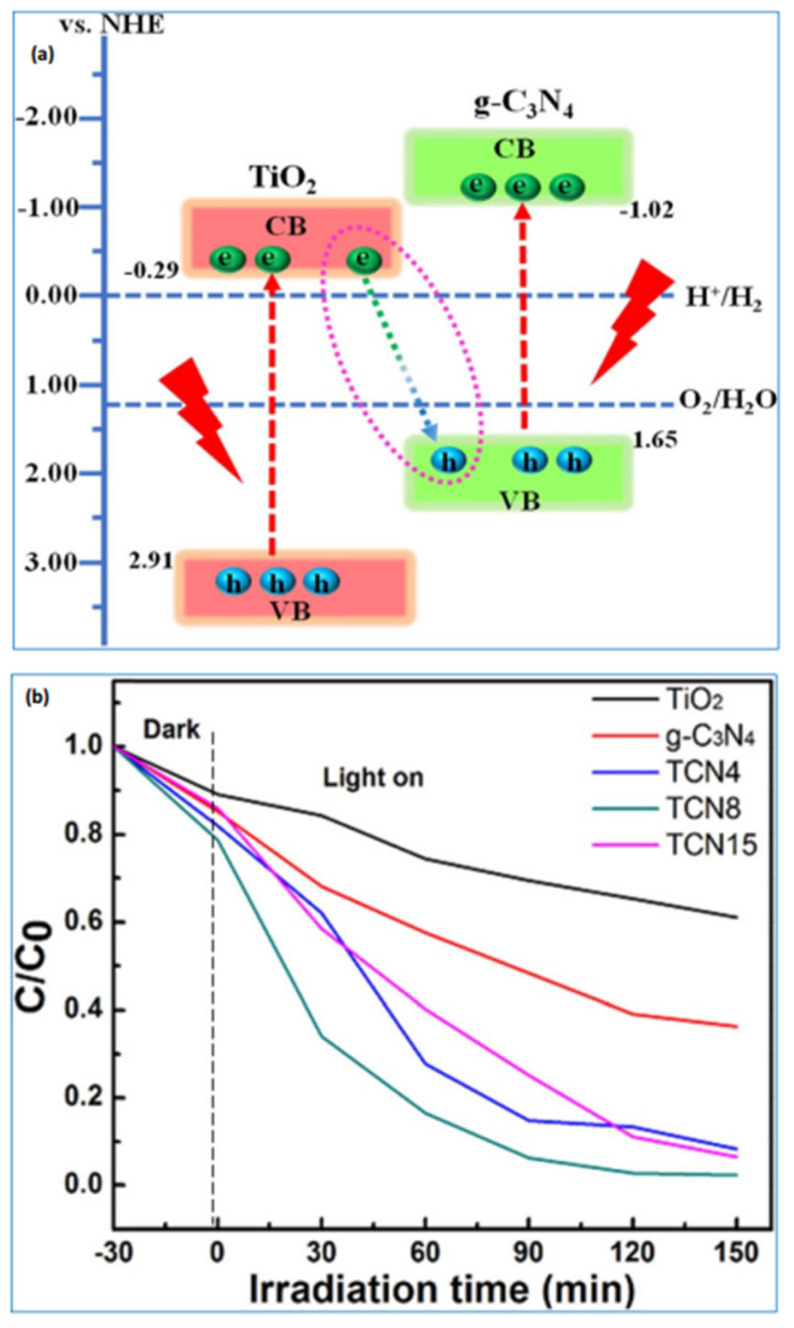
(**a**) Suggested mechanism for methylene blue (MB) degradation using the graphitic carbon nitride (g-C_3_N_4_/TiO_2_) system. (**b**) Photocatalytic degradation efficiency of MB using pure TiO_2_, g-C_3_N_4_, and g-C_3_N_4_/TiO_2_ composites containing 4%, 8%, and 15% by weight of g-C_3_N_4_, referred to as TCN4, TCN8, and TCN15, respectively, under xenon lamp illumination [31]. *Copyright 2019 Springer Nature*.

As seen from Figure 1a TiO_2_ is recognized as a highly effective photocatalyst on its own, and forming a composite with g-C_3_N_4_ can significantly enhance its photocatalytic activity for degrading organic pollutants. Jia et al. synthesized TiO_2_ using the Metal–Organic Framework (MOF) precursor MIL-125 and prepared g-C_3_N_4_ from melamine [28,29]. They fabricated a g-C_3_N_4_/TiO_2_ composite incorporating Z-scheme heterojunctions via a calcination process. This composite was employed for the photocatalytic degradation of methylene blue (MB), demonstrating superior efficiency compared to individual g-C_3_N_4_ or TiO_2_. Among various compositions, the composite containing 8 wt% g-C_3_N_4_ exhibited the highest degradation efficiency (97.7%) within 150 min. Although the photocatalytic activity improved relative to pure g-C_3_N_4_, the overall performance was still considered suboptimal [30,31].

To enhance the heterojunction characteristics and improve photocatalytic efficiency (Figure 1b, TiO_2_ was employed. Its band edge positions (E_CB_ = −0.39 eV, E_VB_ = 3.33 eV) align well with those of g-C_3_N_4_ (E_CB_ = −1.4 eV, E_VB_ = 1.3 eV), facilitating effective electron transfer from g-C_3_N_4_ to La_2_Ti_2_O_7_. This efficient charge separation leads to the generation of more reactive species due to the presence of highly reducible electrons. Consequently, a g-C_3_N_4_/La_2_Ti_2_O_7_ composite photocatalyst was developed, showing excellent performance in the degradation of methylene blue (MB). La_2_Ti_2_O_7_ nanosheets were synthesized via the hydrothermal method, while g-C_3_N_4_ powders were obtained by thermal treatment of melamine in a muffle furnace. The composite was then formed using the wet impregnation technique, achieving enhanced photocatalytic activity, with 90% of MB degraded within 2 h [30].

**Figure 2:**
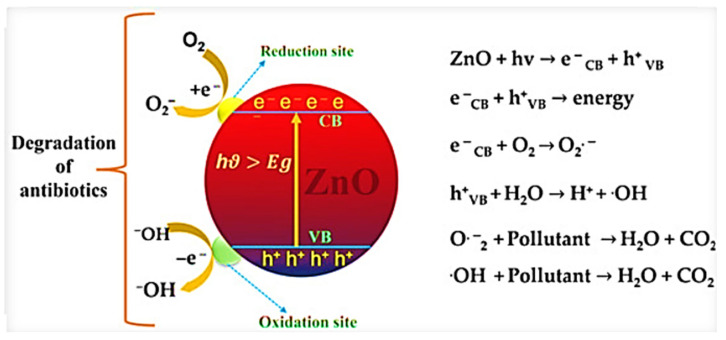
Photocatalysis mechanism of ZnO [36]. *Copyright 2023 MDPI*.

Hermann et al. [32] presented a comprehensive mechanism outlining the photocatalytic oxidation steps involved when using ZnO as a catalyst: (i) pollutants first migrate from the liquid phase to the surface of ZnO nanoparticles, where they are adsorbed; (ii) redox reactions occur during this adsorption, followed by the desorption of the resulting products; and (iii) finally, the degraded pollutants are removed from the surface region. When ZnO is irradiated with UV or solar light possessing photon energy (hv) greater than its bandgap energy (Eg), electrons from the valence band (VB) are excited to the conduction band (CB), leaving behind holes. This process generates electron–hole (e^−^/h^+^) pairs, which migrate to the ZnO surface and initiate redox reactions. Holes react with water or hydroxide ions to form hydroxyl radicals, while electrons react with oxygen to produce superoxide anions that subsequently form hydrogen peroxide. This hydrogen peroxide then further reacts with superoxide radicals to generate more hydroxyl radicals. These highly reactive hydroxyl radicals aggressively attack the adsorbed organic pollutants on the ZnO surface, leading to the formation of intermediate products [33,34], which are ultimately converted into harmless end products such as water (H_2_O), carbon dioxide (CO_2_), and mineral acids. The complete photocatalytic process is illustrated in Figure 2.

The term “degradation of antibiotics” in Figure 2 refers to the photocatalytic breakdown of antibiotic molecules into simpler, less harmful compounds through redox reactions mediated by ZnO under light irradiation [35]. When ZnO absorbs photons with energy greater than its band gap (hν > Eg), electron–hole pairs are generated, with electrons excited to the conduction band (C_B_) and holes remaining in the valence band (V_B_). These charge carriers migrate to the surface of ZnO, where reduction and oxidation processes occur simultaneously. At the reduction sites, photogenerated electrons react with dissolved oxygen to form superoxide radicals (O_2_•^−^), while at the oxidation sites, photogenerated holes react with water molecules or hydroxide ions to produce hydroxyl radicals (•OH). Both O_2_•^−^ and •OH are highly reactive oxygen species (ROS) capable of attacking the molecular structure of antibiotics, leading to cleavage of chemical bonds such as amide, aromatic, and heterocyclic groups. As a result, the complex antibiotic molecules are mineralized stepwise into smaller intermediates and ultimately converted into benign end products such as CO_2_, H_2_O, and inorganic ions. Thus, “degradation of antibiotics” denotes the complete or partial mineralization of antibiotic contaminants in water through ZnO-driven photocatalysis, reducing their toxicity and environmental persistence.

The optical and defect-related properties of TiO_2_, ZnO, and BiVO_4_ can be effectively analyzed using UV–Vis spectroscopy and photoluminescence (PL) techniques (seen in Table 2). UV–Vis measurements, performed through diffuse reflectance spectroscopy (DRS) or transmission methods, reveal that TiO_2_ possesses a bandgap of ~3.0–3.2 eV with an absorption edge around 390 nm, ZnO has a bandgap of ~3.3 eV with an absorption edge near 375 nm, and both materials primarily respond to UV light. In contrast, BiVO_4_, with a bandgap of ~2.4–2.5 eV and an absorption edge around 520 nm, responds to both UV and visible light. Bandgap determinations are typically carried out using Tauc plots, with TiO_2_ showing both indirect and direct transitions, ZnO exhibiting direct transitions, and BiVO_4_ also displaying a direct transition nature. PL studies further provide insights into emission characteristics and defect states. Anatase TiO_2_ exhibits UV (~380 nm) and visible (blue green) emissions originating from band-edge transitions and surface oxygen vacancies, particularly Ti^3+^ states, which are relevant for photocatalytic activity monitoring and defect analysis. ZnO demonstrates strong UV emission (~380 nm) from excitonic recombination and a pronounced green emission (~500–550 nm) from deep-level defects such as zinc vacancies, oxygen vacancies, and interstitials, making it valuable for LED and sensor applications. Monoclinic BiVO_4_ shows a broad visible emission (~500–700 nm) arising mainly from defect states, oxygen vacancies, and V^5+^/V^4+^ centers, which are important for studying charge carrier recombination in photocatalytic processes.

#### DFT Insights: Band Structure, DOS, Charge Density Maps of TiO_2_, ZnO, BiVO_4_

**Figure 3:**
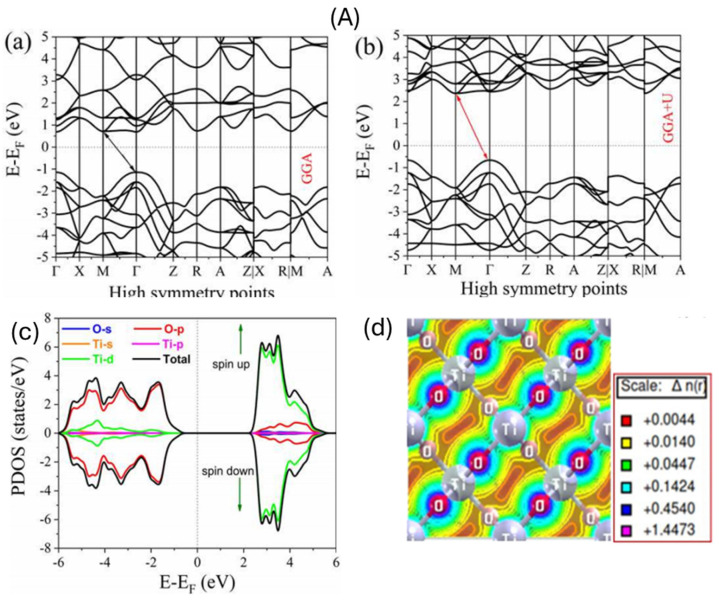

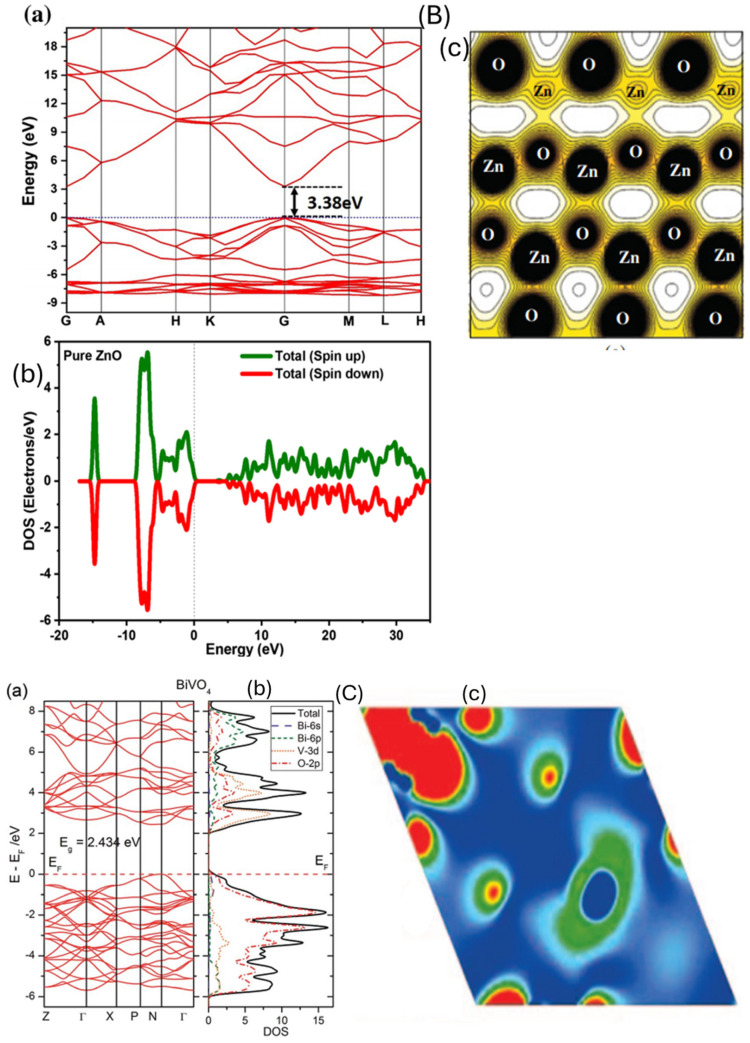
(**A**) Band structures of (**a**) Generalized Gradient Approximation (GGA) pure rutile TiO_2_, (**b**) Generalized Gradient Approximation + correction (GGA + U) pure rutile TiO_2_, (**c**) the total and partial density of states (TDOS and PDOS) of pure rutile TiO_2_ [52]. (**d**) Charge density of TiO_2_ [53]. *Copyright 2025 MDPI* (**B**). (**a**) Band structures of pure ZnO. (**b**)The spin polarized density of states of pure ZnO [54]. *Copyright 2021 Springer.* (**c**) (Color online) Charge density of ZnO [55]. *Copyright 2011 American Physical Society* (**C**). (**a**) Band structures of pure BiVO_4_ (**b**) density of states BiVO_4_ [56]. *Open access.* (**c**) Charge density of BiVO_4_ [57]. *Copyright 2009 American Chemical Society*.

The analysis of the electronic structure, density of states (DOS), and charge density distributions of semiconductor materials such as TiO_2_, ZnO, and BiVO_4_ plays a pivotal role in understanding their optical and electronic properties. These insights are critical for applications in photocatalysis, photovoltaics, and optoelectronics. The data presented in Figure 3A–C can be interpreted comprehensively as follows:

Figure 3A(a–d) focus on the pure rutile phase of TiO_2_. Figure 3A(a) shows the band structure calculated using the Generalized Gradient Approximation (GGA) [44], while Figure 3A(b) illustrates the band structure under the GGA + U correction scheme. The GGA method typically underestimates the band gap of TiO_2_, as seen from the near-zero bandgap nature in the GGA calculation. The valence band (V_B_) is primarily composed of O 2p states, while the conduction band (C_B_) is dominated by Ti 3d states. The valence band maximum (VBM) consists of hybridized O 2p and Ti 3d states. The conduction band minimum (CBM) is mainly Ti 3d. Defects (e.g., oxygen vacancies) introduce mid-gap states, as seen in DFT+U studies [45]. Figure 3A(c) presents the partial density of states (PDOS), revealing the orbital contributions near the Fermi level. The valence band is predominantly composed of O 2p states, while the conduction band is mainly Ti 3d in character. Spin-polarized DOS further indicates symmetrical distribution between spin-up and spin-down states, confirming the non-magnetic nature of the pristine rutile TiO_2_. The charge density distribution in Figure 3A(d) further elucidates the bonding characteristics. It shows high electron density near the O atoms, while Ti atoms exhibit depleted electron density, consistent with the ionic nature of Ti-O bonding. The charge accumulation between Ti and O also suggests some covalent interaction. The color scale quantitatively represents the charge transfer between atoms [46].

Figure 3B(a) illustrates the band structure of pure ZnO, revealing a direct band gap of approximately 3.38 eV at the Γ point, consistent with known experimental values for wurtzite ZnO. This wide band gap positions ZnO as a promising candidate for UV-light-driven photocatalytic and optoelectronic applications. Figure 3B(b) displays the spin-polarized DOS for pure ZnO. Unlike TiO_2_, the asymmetry between spin-up and spin-down states may suggest spin polarization due to intrinsic defects or local magnetic moments, although in ideal stoichiometric ZnO, the spin-up and spin-down states should be symmetric. The valence band is mainly composed of O 2p states, while the conduction band is dominated by Zn 4s states. The charge density map in Figure 3B(c) shows a relatively even distribution between Zn and O atoms, consistent with significant ionic bonding. However, a degree of hybridization indicates partial covalency, which may contribute to the enhanced photocatalytic performance by facilitating charge separation at the interface [47,48]. DFT+U corrections improve bandgap predictions, aligning better with experiments [48]. Therefore, the spin-dependent properties of ZnO are important because any spin polarization, often arising from intrinsic defects or localized magnetic moments, can influence charge carrier dynamics and recombination. This affects photocatalytic efficiency by potentially enhancing charge separation and reactivity. Thus, even subtle spin effects provide insight into defect states and their role in ZnO’s photocatalytic and optoelectronic performance

Bismuth Vanadate (BiVO_4_) has a bandgap of ~2.4–2.5 eV (monoclinic scheelite), making it suitable for visible-light absorption. DFT+U studies show a direct bandgap at Γ-point [49]. Figure 3C(a–c) present the electronic structure of monoclinic BiVO_4_. The band structure shown in Figure 3C(a) exhibits an indirect band gap of approximately 2.434 eV, positioning BiVO_4_ as a visible-light-active photocatalyst. The indirect nature is evident as the VBM and CBM occur at different k-points. The DOS plot in Figure 3C(b) provides orbital-specific insights. The valence band edge is primarily contributed by O 2p states, while the CBM has significant contributions from V 3d orbitals, as well as minor Bi 6p components. This combination enhances the optical absorption in the visible range, making BiVO_4_ suitable for solar-driven applications [50]. The charge density distribution in Figure 3C(c) further confirms the nature of bonding. The charge is highly localized around O atoms, while Bi and V atoms exhibit lower electron densities. The visualization suggests mixed ionic-covalent bonding, with directional electron sharing between V and O atoms, consistent with partial hybridization in VO_4_ tetrahedra [51].

In summary, the comparative analysis across TiO_2_, ZnO, and BiVO_4_ reveals distinct electronic characteristics suitable for various applications. TiO_2_ exhibits an indirect band gap with a strong ionic-covalent character, ZnO shows a wide direct band gap with more ionic bonding, and BiVO_4_ features a visible-light-active indirect band gap with complex hybridization. These electronic properties, as elucidated through DFT-based band structures, DOS, and charge density maps, are foundational in guiding the optimization and application of these materials in energy and environmental technologies.

### 2.2. Solar Energy Conversion

Photoanodes are central to solar energy conversion as they absorb light, generate electron–hole pairs, and drive oxidation reactions. The efficient conversion of solar energy into chemical fuels, such as through photoelectrochemical (PEC) water splitting, relies critically on the performance of photoanodes. These semiconductor electrodes are responsible for absorbing sunlight to generate electron–hole pairs, facilitating the oxidation reaction (e.g., oxygen evolution). Ideal photoanode materials must possess a narrow bandgap for broad visible light absorption, band edges that straddle the water oxidation/reduction potentials, and excellent charge carrier mobility to minimize recombination losses. Among the most studied materials are TiO_2_, ZnO, and BiVO_4_, each offering distinct advantages and limitations [58]. When coupled with TiO_2_, band equilibration drives electron transfer from BiVO_4_ to TiO_2_, enhancing charge separation.

**Figure 4:**
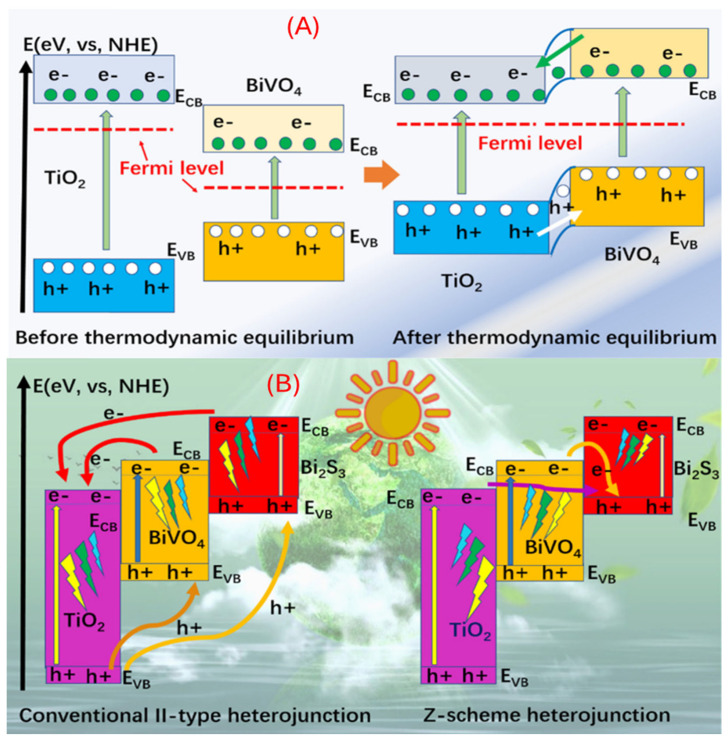
(**A**) The diagram of the energy band before and after TiO_2_ and BiVO_4_ formation heterojunction. (**B**) The charge transfer mechanism for Bi_2_S_3_/BiVO_4_/TiO_2_ [58]. *Copyright 2023 De Gruyter Bill*.

Figure 4A illustrates the energy band configuration of TiO_2_ and BiVO_4_ before and after junction formation. Heterojunction strategies are widely employed to improve performance. In situ growth of Bi_2_S_3_ on BiVO_4_ yields a ternary Bi_2_S_3_/BiVO_4_/TiO_2_ structure with strong photoelectric responses [59,60]. Two carrier-separation pathways have been proposed (Normal Hydrogen Electrode (NHE)): (i) a type-II heterojunction, where electrons cascade from Bi_2_S_3_ → BiVO_4_ → TiO_2_ while holes migrate oppositely, confirmed via PL quenching, photocurrent, EIS, and band-edge measurementss [58,59,61]; and (ii) a Z-scheme mechanism, where electrons in the CBs of TiO_2_/BiVO_4_ recombine with holes in the V_B_ of Bi_2_S_3_, leaving high-energy electrons in Bi_2_S_3_ and holes in BiVO_4_/TiO_2_. The Z-scheme can be distinguished by ESR radical detection, scavenger tests, selective photodeposition, and transient absorption [58,61,62].

The two carrier-separation mechanisms in Bi_2_S_3_/BiVO_4_/TiO_2_ type-II and Z-scheme can be experimentally distinguished using complementary techniques [63]. In a type-II pathway, charge transfer can be confirmed through PL quenching, transient photocurrent, EIS, and band alignment measurements (e.g., UV–Vis DRS, UPS, Mott–Schottky) [62]. In contrast, the Z-scheme mechanism can be demonstrated by radical trapping/ESR studies, scavenger experiments, selective photodeposition of oxidation/reduction products, and transient absorption spectroscopy, which collectively prove retention of high redox potential [58,61].

Performance enhancements further rely on doping, surface passivation, and interface modification, which suppress recombination and extend absorption. Density functional theory (DFT) provides valuable insight into band alignment and carrier dynamics, guiding rational design of heterostructures. As illustrated in Figure 4B, the Bi_2_S_3_/BiVO_4_/TiO_2_ composite demonstrates both type-II and Z-scheme possibilities, with growing evidence favoring the Z-scheme for superior charge separation and photocurrent generation. Below is an enhanced deep dive suitable for publication that brings together recent findings on TiO_2_, ZnO, and BiVO_4_ in solar energy conversion, focusing on their roles as photoanodes, interface engineering, and DFT modelling for energy-level alignment.

#### 2.2.1. Role of TiO_2_, ZnO, and BiVO_4_ as Photoanodes

Metal oxide semiconductors such as TiO_2_, ZnO, and BiVO_4_ are pivotal in next-generation solar conversion devices. TiO_2_ remains the gold standard in both dye-sensitized solar cells (DSSCs) and n–i–p perovskite solar cells (PSCs) due to its optimal band alignment (~–4.0 eV conduction band), transparency, and chemical stability [64,65]. ZnO possesses similar energy alignment to TiO_2_, with the added benefit of higher electron mobility. However, its thermal instability above ~70 °C can degrade adjacent perovskites, demanding surface passivation strategies [66]. BiVO_4_ serves as a robust photoanode mainly in photoelectrochemical (PEC) water-splitting due to its ~2.4 eV bandgap for visible light absorption; compositing with ZnO is shown to promote charge separation and hole transport via cocatalysts like Fe-ZIF-8 [67].

#### 2.2.2. Interface Engineering

Achieving high efficiency and stability in PSCs and PEC systems hinges on atomic-scale interface engineering between oxide layers and light absorbers: Potassium trifluoromethyl sulfonate molecules crosslink TiO_2_ and perovskite, mitigating trap states at oxygen and titanium vacancies. This strategy has led to champion PCEs exceeding 25% and outstanding operational stability (>81% retainment after 1000 h) [67]. Recent work with 4-chloro-3-sulfamoylbenzoic acid (CSBA) on TiO_2_ highlights how directional self-assembled monolayers reduce the conduction band offset from 0.40 to 0.13 eV, enabling certified PCEs of 25.3% on small-area devices and 24.2% on 1 cm^2^ modules [65]. Combining atomic-layer deposited TiO_2_ with spin-coated SnO_2_ produces a bilayer ETL with enhanced grain size, reduced hysteresis, and high current densities. These devices reach PCEs up to 17.6% with stable operation [68]. Incorporation of ammonium chloride into SnO_2_ ETL optimizes band alignment and passivates surface defects, extending device stability to over 2400 h (>95% efficiency retention) [69]. Moreover, engineering specific crystal facets such as (111)-oriented cubic SnO_2_ has yielded PCEs of 20.3% on triple-cation perovskites, retaining over 81% performance after 480 h [70].

Doping ZnO nanorods can curb electronic vacancies and boost mobility for flexible PSC applications [71], while ZnO/ZnS core–shell nanostructures eliminate hydroxyl-induced trap states in PeLEDs, raising EQE from 1.4% to 8.9% [72]. In PEC systems, integrating Fe-ZIF-8 on BiVO_4_/ZnO builds an internal electric field across the heterojunction, increasing charge separation (to ~80%) and PEC current density to 4.14 mA cm^−2^ [67].

### 2.3. DFT Modeling for Interface and Energy Level Optimization

Density Functional Theory (DFT) has become essential for engineering oxide–absorber interfaces with precision: DFT combined with photoemission spectroscopy confirmed that directional SAMs (e.g., CSBA) adjust TiO_2_/perovskite C_B_ offsets reducing them from 0.40 eV to 0.13 eV optimizing electron injection while maintaining suitable V-OC [65]. First-principles modeling uncovers atomically crisp PbO-capped MAPbI_3_/TiO_2_ interfaces that form metallic 2D “zipper” layers from strain. This unique interface doubles the charge extraction rate [73]. DFT and UPS investigations demonstrate that incorporated Ti_3_C_2_T_x_ MXenes shift the work functions of both perovskite and TiO_2_ (~0.35 eV), establishing stronger built-in fields and enhancing charge transport, while passivating Pb surface traps [74]. DFT analyses elucidate how TiO_2_-SnO_2_ amalgam ETLs align well with perovskite energy bands, yielding high electron mobility due to minimized interfacial trap states, which manifests in devices with 24.6% conversion efficiency (PCE). Perovskite solar cells (PSCs) consistently exceed 25% PCE, while BiVO_4_ and ZnO show strong promise in PEC and flexible applications. DFT studies are increasingly predicting new interface phenomena such as interface metallicity and dipole-induced internal fields enabling targeted experimental validation. Future directions lie in expanding 2D-material interlayers, dopant engineering, and strain-modulated heterointerfaces, with DFT continually guiding this path towards commercial-grade solar technologies.

### 2.4. Energy Storage

The rapid growth in portable electronics, electric vehicles, and grid-scale energy storage has intensified the demand for efficient, stable, and high-capacity energy storage systems. Among various materials investigated, transition metal oxides (TMOs) have emerged as promising candidates for both lithium-ion batteries (LIBs) and supercapacitors, due to their rich redox chemistry, high theoretical capacities, and structural diversity [75,76].

**Figure 5:**
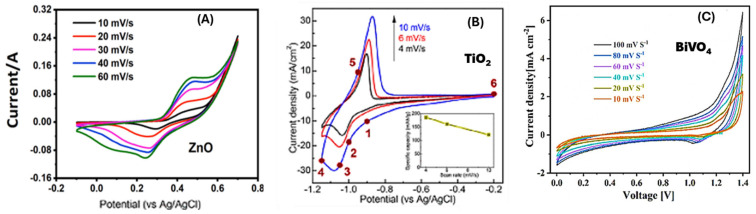
CV curves at different current densities of (**A**) ZnO [77]. *Copyright 2022 Elsevier.* (**B**) TiO_2_. *Copyright 2023 MDPI.* (**C**) BiVO_4_ [78]. *Copyright 2025 Springer*.

Figure 5 presents CV measurements, a fundamental electrochemical technique where the current response of a material is measured while the potential between the working and reference electrodes is swept back and forth. This is used to study the charge storage behavior and electrochemical properties of materials, often for applications like supercapacitors or electrocatalysts.

The CV curves shown in Figure 5A for ZnO are relatively rectangular, especially at lower scan rates (e.g., 10 mV/s). This is the characteristic shape of an Electric Double-Layer Capacitor (EDLC). In EDLCs, energy is stored electrostatically by the adsorption of ions from the electrolyte onto the electrode surface, with no faradaic (redox) reactions involved. As the scan rate increases from 10 to 60 mV/s, the curves maintain their rectangular shape but the current response increases. This is expected because a higher scan rate drives ions to the surface at a faster rate, resulting in a higher current (i = C × v/dt, where dv/dt is the scan rate). The lack of significant distortion at higher scan rates suggests good rate capability and efficient ion transport within the ZnO electrode structure. Upon closer inspection, there might be very small, broad humps in the curves, indicating a minor contribution from pseudocapacitance. This suggests that in addition to the primary EDLC behavior, some reversible surface faradaic reactions are also occurring.

The CV curves in Figure 5B for TiO_2_ show distinct, broad, and symmetric oxidation and reduction peaks. This is the classic signature of pseudocapacitive charge storage. Pseudocapacitance involves fast, reversible faradaic reactions (redox reactions) on or near the surface of the material.TiO_2_ primarily stores charge through surface redox reactions, making it a pseudocapacitive material. The well-defined peaks and their evolution with scan rate are clear evidence of this mechanism.

The CV curves in Figure 5C for BiVO_4_ are drastically different from the other two. They feature very large, sharp, and asymmetric redox peaks. The anodic (positive current) peak is much larger and sharper than the cathodic (negative current) peak. This shape is highly characteristic of battery-like or diffusion-controlled faradaic behavior. This means charge is stored through bulk redox reactions that involve phase changes and are often limited by the diffusion of ions into the material’s structure. Thus, BiVO_4_ exhibits primarily battery-type behavior, where charge storage is dominated by diffusion-controlled faradaic reactions in the bulk material, not just on the surface.

The integration of MXene and graphene architectures with TiO_2_, ZnO, and BiVO_4_ significantly mitigates their inherent limitations such as poor conductivity, volume changes, and sluggish ion transport while unlocking superior electrochemical performance. For example, ZnO nanorods anchored onto three-dimensional graphene frameworks created a conductive, flexible matrix that buffered lithiation-induced stress and delivered a remarkable initial discharge capacity of 1583 mAh g^−1^ at 200 mA g^−1^, retaining 886 mAh g^−1^ after 100 cycles [79]. In another compelling instance, a freestanding ZnO/graphene/CNT ternary composite achieved a high first discharge capacity of 1503 mAh g^−1^ and maintained a reversible 620 mAh g^−1^ after 100 cycles, thanks to the conducting network inhibiting ZnO nanoparticle aggregation and accommodating volume changes [80]. For BiVO_4_-based electrodes, reduction-graphene-oxide hybrids demonstrated excellent supercapacitor performance, with energy density reaching 33 Wh kg^−1^ and capacitance retention of 81% after 8000 cycles [81]. While direct reports of TiO_2_/MXene or TiO_2_/graphene hybrids remain underexplored in energy-storage systems, MXenes, in general, exhibit extraordinary conductivity and tunable surface chemistry, enabling high volumetric capacitance (e.g., up to 1500 F cm^−3^ for Ti_3_C_2_T_x_ hydrogels) and promising composite platforms when interfaced with metal oxides. These examples underscore that MXene and graphene composites are not tangential but fundamentally complementary: they create conductive, mechanically flexible networks that straddle and amplify the intrinsic merits of TiO_2_, ZnO, and BiVO_4_ yielding electrode materials with enhanced capacity, rate performance, cyclic stability, and energy-storage versatility.

#### 2.4.1. Transition Metal Oxides (e.g., MnO_2_, Fe_2_O_3_)

TMOs such as MnO_2_, Fe_2_O_3_, Co_3_O_4_, and NiO have been extensively explored due to their favorable redox behavior, availability, and environmental benignity. For example, MnO_2_ offers a theoretical capacity of ~1230 mAh/g, arising from multivalent redox reactions (Mn^4+^/Mn^3+^/Mn^2+^), and exhibits pseudocapacitive behavior advantageous in supercapacitor applications [82,83]. Fe_2_O_3_, with a theoretical capacity of 1007 mAh/g, also undergoes reversible conversion reactions (Fe^3+^ + 3e^−^ ↔ Fe^0^), but often suffers from volume changes and poor conductivity [84]. Recent advances focus on nanoscale engineering (e.g., hollow spheres, nanowires), heterostructures, and doping strategies to overcome limitations such as low intrinsic conductivity and capacity fading. For example, introducing conductive carbon matrices or combining TMOs with graphene or MXenes has significantly improved their rate capabilities and cycling stability [85,86]

#### 2.4.2. Experimental Cycling/Stability Studies

The electrochemical performance of TMOs is typically evaluated through galvanostatic charge–discharge (GCD), cyclic voltammetry (CV), and electrochemical impedance spectroscopy (EIS). Key performance metrics include specific capacity, capacity retention, C-rate performance, and cycling stability. For instance, MnO_2_-based electrodes have shown high initial capacities, but capacity fading is often observed due to structural degradation and polysulfide dissolution. Strategies such as core–shell architectures or 3D porous scaffolds have been employed to buffer volume expansion and improve cycling stability [87]. In a recent study, Fe_2_O_3_ nanoparticles embedded in carbon nanofibers retained over 92% capacity after 1000 cycles at 1 A/g, highlighting the significance of mechanical confinement and conductive networks in long-term operation [88].

#### 2.4.3. Density Functional Theory (DFT) Analysis of Ion Diffusion and Redox Potentials

Density functional theory (DFT) calculations are indispensable for evaluating the phase stability of metal oxide electrodes like TiO_2_, ZnO, and BiVO_4_ at varying levels of ion intercalation, a critical factor dictating structural integrity, voltage profiles, and capacity retention in batteries. By computing the formation energy of numerous intermediate configurations and constructing convex hull diagrams, researchers can identify stable phases, metastable pathways, and deleterious phase separations that lead to mechanical degradation and capacity fade. For instance, recent studies on anatase TiO_2_ have used DFT to unravel the thermodynamic origins of its biphasic transition during lithiation, guiding surface engineering strategies for improved stability [89]. Similarly, for ZnO, DFT analyses reveal how conversion reactions and alloying mechanisms lead to large volume changes, prompting investigations into nanocomposite designs to mitigate pulverization. In the case of multivalent batteries, DFT screening of BiVO_4_ polymorphs for Mg^2+^ insertion helps predict stable host structures and intercalation voltages, accelerating the discovery of durable cathodes [89]. Thus, phase stability calculations provide a fundamental roadmap for tailoring the electrochemical properties and longevity of next-generation oxide-based battery materials.

DFT calculations play a critical role in elucidating ion diffusion pathways, redox potentials, and electronic structure modifications in TMOs at the atomic level. The migration barrier energy (E_m_) for Li^+^/Na^+^ diffusion is often calculated using the nudged elastic band (NEB) method, providing insight into rate capabilities [90]. For example, DFT studies have shown that α-MnO_2_ offers 1D tunnels suitable for Li^+^ insertion, with relatively low diffusion barriers (~0.3 eV), while β-MnO_2_, due to its denser structure, presents higher barriers (~0.6–0.8 eV) [91]. Similarly, Fe_2_O_3_ shows moderate barriers (~0.45 eV) for Li^+^ diffusion, which can be reduced by oxygen vacancies or doping with Co/Ni [92]. The calculated redox potentials from DFT+U methods are consistent with experimental observations. For instance, the Fe^3+^/Fe^2+^ redox couple in Fe_2_O_3_ appears near 1.5–1.6 V vs. Li/Li^+^, while Mn-based systems (Mn^4+^/Mn^3+^) operate at ~3.0 V. By analyzing the projected density of states (PDOS), DFT reveals the participation of metal 3d and O 2p orbitals in charge storage, and how hybridization affects conductivity and reversibility [93].Advanced machine-learning-augmented DFT methods have also been employed to predict ion intercalation behavior and guide the discovery of novel TMO compositions with enhanced performance [94].

## 3. Challenges and Future Prospects

### 3.1. Challenges

Despite decades of progress, TiO_2_, ZnO, and BiVO_4_ still face intertwined bottlenecks that theory and experiment often capture only in parts. For BiVO_4_, the fundamental limits of a relatively large band gap, sluggish OER interfacial kinetics, and short carrier diffusion lengths persist even when cocatalysts or dopants are used, and these device-level losses are not yet predicted reliably by routine DFT workflows [95]. Bridging studies show that surface states and near-surface defect chemistry dominate charge transfer, but operando probes (XPS/XAS, Raman, transient spectroscopy) reveal dynamic restructuring under bias/illumination that standard static calculations rarely include, leading to model–experiment mismatch [96,97]. For ZnO, defect/dopant complexity (e.g., N, Mg/B/N codoping) strongly tunes absorption and carrier lifetimes, yet the exact roles of compensating defects and polaronic trapping remain difficult to parameterize beyond idealized supercells [98,99]. Even for “mature” TiO_2_, activity hinges on specific facets, metal clusters, and transient active sites; first-principles predictions often underrepresent these operando-formed ensembles, while ex situ characterizations can miss light- and potential-driven transformations [100,101]. At a methods level, band-gap errors, missing excitonic/solvation effects, and inadequate treatment of electrochemical boundary conditions (pH, potential, ion pairing) limit quantitative agreement and transferability across synthesis routes and device architectures [102].

### 3.2. Future Prospects

Converging operando/perturbative experiments with advanced theory offers a practical pathway forward: couple in situ XPS/XAS/Raman and ultrafast spectroscopy to parameterize surface states, catalyst reconstruction, and charge-transfer kinetics in the same electrolyte/device geometry used for computation-aware validation [96,97]. On the theory side, wider adoption of hybrid/meta-GGA functionals and beyond-DFT corrections (where affordable) should improve band alignment and defect energetics, while emerging workflows fuse DFT descriptors with machine learning to rapidly screen dopants/heterojunctions and then down-select targets for synthesis [102]. For BiVO_4_, targeted surface engineering (e.g., gradient doping, oxygen vacancy control, and OER-specific cocatalyst architectures) guided by operando benchmarks can mitigate interfacial overpotentials and extend carrier lifetimes [103]. In ZnO, defect-aware design validated by DFT-informed dopant selection and verified under illumination can shift absorption into the visible while suppressing recombination; recent reports illustrate how N and multi-element doping, as well as oxide/carbon hybrids, deliver this synergy [98]. For TiO_2_, facet engineering combined with controlled noble-metal ensembles (from clusters to single atoms) characterized operando can rationally stabilize active motifs for H**_2_** evolution and pollutant oxidation [100,104]. Overall, establishing closed-loop pipelines (i) theory-proposed structures → (ii) synthesis with in situ verification of the intended motifs → (iii) device-level testing under realistic bias/illumination → (iv) model updating with operando data will be key to truly “bridging” experiment and theory for these workhorse oxides [96,97].

## 4. Conclusions

The synergy between density functional theory (DFT) and experimental investigations has significantly advanced the understanding and optimization of TiO_2_, ZnO, and BiVO_4_ for renewable energy applications, yet challenges remain in fully bridging theory with real-world performance. Experimental–theoretical studies reveal that heterojunctions, doping, and defect engineering enhance light absorption, charge separation, and catalytic activity, with composites such as TiO_2_/g-C_3_N_4_, ZnO-based hybrids, and BiVO_4_ heterostructures demonstrating notable improvements in photocatalysis, solar energy conversion, and energy storage. However, persistent bottlenecks include limited visible light absorption in TiO_2_, photocorrosion in ZnO, and poor charge transport in BiVO_4_, with theory often underrepresenting the dynamic surface and defect behaviors observed under operando conditions. Standard DFT methods, despite their utility in band structure and DOS analysis, struggle with bandgap errors, excitonic effects, and electrochemical boundary conditions, limiting predictive accuracy. To overcome these issues, future research should integrate operando probes (XPS/XAS, Raman, ultrafast spectroscopy) with advanced DFT and machine learning to capture dynamic charge-transfer processes and defect energetics under realistic device conditions. Targeted strategies such as oxygen vacancy control and gradient doping in BiVO_4_, defect-aware dopant engineering in ZnO, and facet or single-atom ensemble tuning in TiO_2_ show strong promise for performance enhancement. Ultimately, establishing closed-loop pipelines that connect theory-driven design, synthesis, operando validation, and iterative modeling will enable the rational development of efficient, stable, and scalable oxide-based systems for photocatalysis, photoelectrochemistry, and energy storage, thereby making the “bridge” between experiment and theory both functional and transformative in advancing renewable energy technologies.

## Figures and Tables

**Table 1:** The application of selective metal oxide.

Materials	Light Absorption	Charge Transport	Surface Defects	Application	Ref.
TiO_2_	Primarily absorbs UV light due to a wide bandgap (~3.2 eV). Visible light absorption can be enhanced through doping or forming heterojunctions.	Exhibits rapid electron–hole recombination; strategies like doping and heterojunction formation improve charge separation.	Surface defects, such as oxygen vacancies, can trap charge carriers, influencing photocatalytic activity.	Water splitting, pollutant degradation.	[21]
ZnO	Absorbs UV light with a bandgap of ~3.3 eV. Visible light activity can be achieved through defect engineering and composite formation.	Similarly to TiO_2_, it suffers from rapid recombination; heterostructures can enhance charge separation.	Oxygen vacancies and other defects can introduce mid-gap states, affecting photocatalytic efficiency.	Pollutant degradation, water splitting.	[22,23,24]
BiVO_4_	Narrower bandgap (~2.4 eV) allows visible light absorption.	Limited by short hole diffusion length (~70 nm) and poor electron mobility	Oxygen vacancies and surface states can enhance or suppress performance depending on concentration	Photocatalytic water splitting, photoelectrochemical (PEC) cells.	[25,26,27]

**Table 2:** Experimental techniques: UV-Vis, PL, XPS, etc.

Experimental Uv-Vis Potential of TiO_2_, ZnO, and BiVO_4_						
Material	Bandgap (eV)	Absorption Edge	Light Response	UV-Vis Technique	Tauc Type	Ref.
TiO_2_	~3.0–3.2	~390 nm	UV only	DRS/Transmission	Indirect/Direct	[37]
ZnO	~3.3	~375 nm	UV only	DRS/Transmission	Direct	[38]
BiVO_4_	~2.4–2.5	~520 nm	UV + Visible	DRS	Direct	[39]
Experimental Photoluminescence (PL) Potential of TiO_2_, ZnO, and BiVO_4_						
TiO_2_ (Anatase)	~3.2	UV (~380 nm) and visible (blue-green)	Band-edge and surface oxygen vacancies	(O vacancy, Ti^3+^ states)	Photocatalysis monitoring, defect analysis	[40,41]
ZnO	~3.3	Strong UV (~380 nm) and visible (green, ~500–550 nm)	Excitonic and deep-level emissions	Zn vacancies, O vacancies, interstitials)	LED devices, sensors, defect studies	[42]
BiVO_4_ (Monoclinic)	~2.4	Broad visible (~500–700 nm)	Defect states, oxygen vacancies, charge carrier trapping	Yes (mainly V^5+^/V^4+^ centers, O vacancies)	Photocatalytic charge recombination study	[26,43]

## Data Availability

No new data were created or analyzed in this study. Data sharing is not applicable to this article.
